# Role of Dystrophin in Airway Smooth Muscle Phenotype, Contraction and Lung Function

**DOI:** 10.1371/journal.pone.0102737

**Published:** 2014-07-23

**Authors:** Pawan Sharma, Sujata Basu, Richard W. Mitchell, Gerald L. Stelmack, Judy E. Anderson, Andrew J. Halayko

**Affiliations:** 1 Department of Physiology and Pathophysiology, University of Manitoba, Winnipeg, Manitoba, Canada; 2 Department of Internal Medicine, University of Manitoba, Winnipeg, Manitoba, Canada; 3 Department of Pediatrics and Child Health, University of Manitoba, Winnipeg, Manitoba, Canada; 4 Section of Respiratory Disease, University of Manitoba, Winnipeg, Manitoba, Canada; 5 CIHR National Training Program in Allergy and Asthma, University of Manitoba, Winnipeg, Manitoba, Canada; 6 Biology of Breathing Group, Manitoba Institute of Child Health, Winnipeg, Manitoba, Canada; 7 Department of Biological Sciences, University of Manitoba, Winnipeg, Manitoba, Canada; Rutgers University -New Jersey Medical School, United States of America

## Abstract

Dystrophin links the transmembrane dystrophin-glycoprotein complex to the actin cytoskeleton. We have shown that dystrophin-glycoprotein complex subunits are markers for airway smooth muscle phenotype maturation and together with caveolin-1, play an important role in calcium homeostasis. We tested if dystrophin affects phenotype maturation, tracheal contraction and lung physiology. We used dystrophin deficient Golden Retriever dogs (GRMD) and *mdx* mice *vs* healthy control animals in our approach. We found significant reduction of contractile protein markers: smooth muscle myosin heavy chain (smMHC) and calponin and reduced Ca^2+^ response to contractile agonist in dystrophin deficient cells. Immunocytochemistry revealed reduced stress fibers and number of smMHC positive cells in dystrophin-deficient cells, when compared to control. Immunoblot analysis of Akt1, GSK3β and mTOR phosphorylation further revealed that downstream PI3K signaling, which is essential for phenotype maturation, was suppressed in dystrophin deficient cell cultures. Tracheal rings from *mdx* mice showed significant reduction in the isometric contraction to methacholine (MCh) when compared to genetic control BL10ScSnJ mice (wild-type). *In vivo* lung function studies using a small animal ventilator revealed a significant reduction in peak airway resistance induced by maximum concentrations of inhaled MCh in *mdx* mice, while there was no change in other lung function parameters. These data show that the lack of dystrophin is associated with a concomitant suppression of ASM cell phenotype maturation *in vitro*, ASM contraction *ex vivo* and lung function *in vivo*, indicating that a linkage between the DGC and the actin cytoskeleton *via* dystrophin is a determinant of the phenotype and functional properties of ASM.

## Introduction

Dystrophin is a large gene (2.2 Mb) gene, which encodes a 427-kDa protein that has an N-terminal actin binding domain, a large central rod domain, a cysteine-rich region, and a C-terminal domain [1], [2]. It is an intracellular protein and a member of dystrophin-glycoprotein complex (DGC) that links the extracellular matrix (ECM) to the underlying actin cytoskeleton [3]. In adult skeletal muscles dystrophin is located at the sarcolemma in connection with the costameric lattice at Z- and M-lines of peripheral sarcomeres [4]. Dystrophin is also present in smooth muscle where it provides a strong link between ECM and actin cytoskeleton. Further, it is distributed in an alternate position with the adherens junction protein vinculin and colocalize with the lipid raft protein caveolin-1 in a rib like manner-arranged parallel to the long axis of the cell [5]–[7].

Lack of dystrophin results in a progressive skeletal muscle wasting disease called Duchenne muscular dystrophy (DMD) [8], [9]. DMD muscle fibers are fragile and leaky [10], [11] and this permeability is made worse by mechanical stress which results in muscle degeneration and a strong inflammatory response followed by severe weakness, fibrosis and atrophy [12], [13]. Lack of dystrophin is also associated with smooth muscle abnormalities in the gastrointestinal tract [14], [15]. Two animal models of DMD which are extensively used to study the pathophysiology and muscle function are **i)** *mdx* mice: caused by a nonsense mutation in exon 23 of the dystrophin gene C57BL10 mice leading to a loss of dystrophin protein expression [8], [16], [17]. Unlike DMD patients, the *mdx* mouse appears to have mild “clinical” manifestation of dystrophy and the *mdx* mouse rarely lives past two years of age while wild-type mice live two and a half to three years [18]–[20]. The muscle phenotype of *mdx* mice is similar to DMD, except for the severity; the damage is more extensive in DMD and has greater functional consequences. Therefore, both *mdx* muscle and DMD muscle show disease progression, although at different rates. *Mdx* muscles have been shown to generate less twitch and tetanic force per cross-sectional area than muscles in wild-type mice [21], [22] and **ii)** The canine-X linked muscular dystrophy (CXMD): caused by a point mutation in canine dystrophin gene resulting in loss of dystrophin transcript and the protein [23], [24]. Golden retriever muscular dystrophy (GRMD) dog is the closest model to DMD in phenotype and histopathology [25]–[28]. These muscles have a complete absence of dystrophin, and show early muscle degeneration. Dogs lose mobility and die by one year of age from respiratory failure. It is considered as an ideal model to study DMD, however, because of the cost and temporal issues associated with using the dog model, most researchers opt to use the mouse model [23], [29]–[32].

Dystrophin is thought to transfer lateral forces from the sarcomeres, to the extracellular matrix, and ultimately, to the tendon [33], [34]. The absence of dystrophin leads to profound reductions in the DGC at the sarcolemma [35]. The lack of structural support at the sarcolemma leaves muscle atrophic, weaker and more susceptible to contraction-induced injury [25], [36]–[41]. Loss in skeletal and cardiac muscle function in a dystrophin deficient animal has been studied and well described but studies in airway smooth muscle *per se* are lacking. We hypothesize that dystrophin has a key role in airway smooth muscle phenotype *in vitro* and also participates in airway smooth muscle contraction and determination of lung function *in vivo.* Taken together, we found that dystrophin is a key determinant of airway smooth muscle phenotype and function.

## Materials and Methods

### Chemicals and reagents

Horseradish peroxidase (HRP)-conjugated goat anti-mouse IgG, HRP-conjugated goat anti-rabbit IgG, and primary antibodies were obtained from the following sources: mouse monoclonal anti-caveolin-1 (BD Transduction Labs, 1 in 1000), mouse monoclonal anti-β-dystroglycan (Novocastra: NCL-b-DG, 1 in 100), mouse monoclonal anti-dystrophin (Chemicon, MAB1692, 1 in 50–200), mouse monoclonal anti-smMHC (1 in 200) and mouse monoclonal anti-calponin (1 in 1000), β-actin (1 in 1000) were obtained from Sigma-Aldrich, St. Louis, MO USA. Rabbit monoclonal anti-phospho Akt1 (Thr 308), rabbit polyclonal anti-phospho-(Ser9/21)-GSK-3 antibody, rabbit anti-phospho-mTOR (Ser2448), total anti-Akt1, GSK3 and mTOR (Cell Signaling Technology, Beverly, MA, USA, 1 in 1000). FITC- conjugated secondary antibodies were from Jackson ImmunoResearch Laboratories. Texas Red-X Phalloidin (T7471) was obtained from Molecular Probes. Cell culture media (DMEM and Ham’s F12) and supplements (fetal bovine serum, ITS-A, penicillin and streptomycin) were obtained from Invitrogen. All other chemicals were of analytical grade.

### Animals used

Experiments were conducted according to the guidelines of the Canadian Council on Animal Care. The protocol was approved by the Committee on the Ethics of Animal Experiments of the University of Manitoba (Number: 09-041). All surgery was performed under sodium pentobarbital anesthesia, and all efforts were made to minimize suffering. All animals were given standard chow and maintained on a 12 h dark and light cycle within the animal facility. *Mdx* mice (dystrophin deficient) and wild-type littermates were obtained under approved protocol number F07-008, held by Dr. Judy Anderson, Faculty of Biological Sciences, University of Manitoba [42], [43]. Canine airway smooth muscle cells were cultured from trachealis muscle of golden retriever muscular dystrophy (GRMD) or healthy littermates [26]–[28]. Canine tissues were obtained from Dr. MK Childers, Wake Forest University Health Sciences, Winston-Salem, NC, USA.

### Primary canine airway smooth muscle cell culture

Primary airway myocytes for cell culture were obtained from dissociated canine as previously described [44]. Cells were plated onto 100 mm culture dishes or pre-cleaned sterile cover slips placed in 6 well culture clusters and grown to confluence using Dulbecco’s modified eagle medium (DMEM) supplemented with 10% fetal bovine serum. At confluence, myocytes were serum deprived a further 7–10 days using Ham’s F12 medium supplemented with insulin, transferrin and selenium (ITS-A, 1%) to induce a contractile phenotype. Cultures were maintained in a humidified chamber at 37°C/5% CO_2_ and all media contained both 100-units/mL penicillin G and 100 µg/mL streptomycin sulfate.

### Preparation of protein lysates from canine airway smooth muscle cells

Primary airway smooth muscle cells from canine trachealis were lysed in ice cold in RIPA buffer (composition: 40 mM Tris, 150 mM NaCl, 1% IgepalCA-630, 1% deoxycholic acid, 1 mM NaF, 5 mM β-glycerophosphate, 1 mM Na_3_VO4, 10 µg/ml aprotinin, 10 µg/ml leupeptin, 7 µg/ml pepstatin A, 1 mM PMSF, pH 8.0). The lysate was transferred to 1.5 ml plastic tube, centrifuged (760×g, 5 min) and the supernatant stored at −20°C for subsequent protein assay and immunoblot analyses.

### Preparation of lung lysates from mdx and wild-type mice

Mouse lungs were cut into small pieces and approximately half of the lungs were preserved in 200 µl of lysis buffer (composition: 40 mM Tris, 150 mM NaCl, 1% IgepalCA-630, 1% deoxycholic acid, 1 mM NaF, 5 mM β-glycerophosphate, 1 mM Na_3_VO_4_, 10 µg/ml aprotinin, 10 µg/ml leupeptin, 7 µg/ml pepstatin A, 1 mM PMSF, pH 8.0) and stored at –20°C for protein analysis. Frozen lung tissues in the lysis buffer were slowly thawed in ice and were transferred into 5 mL tubes for homogenization using a polytron. The lysate was transferred to 1.5 ml plastic tube, centrifuged (760×g, 5 min) and the supernatant stored at −20°C for subsequent protein assay and immunoblot analyses.

### Immunoblotting

Protein content in supernatant samples was determined using the BioRad protein assay with bovine serum albumin as a reference (BioRad, Hercules, CA). Immunoblotting was performed using standard techniques [45]. Briefly, after reconstituting samples in denaturing buffer, 18–25 µg protein was loaded per lane and size-separated electrophoretically under reducing conditions using SDS-polyacrylamide gels. Thereafter proteins were electro-blotted onto nitrocellulose membranes, which were subsequently blocked with 5% w/v skim milk in Tris Buffered Saline (TBS) (composition: 10 mM Tris HCl, pH 8.0, 150 mM NaCl) with (0.2%) or without Tween-20. Blocked membranes were incubated with primary antibodies (dilutions described above) in TBS containing 1% w/v skim milk with (0.2%) or without Tween-20. The membranes were developed by subsequent incubation with HRP-conjugated secondary antibody, and then visualized on photographic film using enhanced chemiluminescence reagents (Amersham, Buckinghamshire, UK). β-actin was used to correct for equal loading of all samples. Densitometry and quantification of the relative protein abundance was performed using the Epson Perfection 4180 Station using either TotalLab TL100 software (Nonlinear Dynamics, Durham, NC) or a gel documentation system (AlphaEaseFC, Alpha Innotech Corporation, San Leandro, CA).

### Immunofluorescence microscopy

Immunofluorescence microscopy was performed using standard techniques described previously [7], [46]. Canine airway smooth muscle cells were plated onto pre-cleared glass coverslips in 6-well culture dishes. Cells were fixed for 15 minutes at 4°C in CB buffer containing 3% paraformaldehyde (PFA). Cells were then permeabilized by incubation for 5 minutes at 4°C in CB buffer containing 3% PFA and 0.3% Triton X-100. For immunofluorescence microscopy, fixed cells were first blocked for 2 hours at room temperature in cyto-TBS buffer containing 1% BSA and 2% normal donkey serum. Incubation with primary antibodies occurred overnight at 4°C in cyto-TBST using anti-smMHC (1∶200), or anti-dystrophin antibody (1∶50). For negative controls, samples were incubated with either isotype-matched mouse IgG or rabbit antiserum. Actin fibers were stained with Texas Red (TxR)- conjugated phalloidin antibody. Incubation with FITC-conjugated secondary antibodies was for 2 hours at room temperature in cyto-TBST. Coverslips were mounted using ProLong antifade medium (Molecular Probes, Inc. USA). Fluorescent imaging was performed by capturing a mid-cell section of 0.3 µm focal depth using an Olympus LX-70 FluoView Confocal Laser Scanning Microscope (Olympus America Inc, Melville, N.Y.) equipped with a 40x objective.

### Transmission electron microscopy (TEM)

The ultrastructure of intact mouse trachea was assessed as described previously with slight modification [46], [47]. Specimens consisting of atleast 4–5 cartilage rings with intact trachealis were prepared from the cervical segments using a sharp scalpel. Specimens were washed once with fresh Krebs-Henseleit solution (KH; 117.5 mM NaCl, 5.6 mM KCl, 1.18 mM MgSO_4_, 2.5 mM CaCl_2_, 1.28 mM NaH_2_PO_4_, 25 mM NaHCO_3_, and 5.55 mM D-glucose, gassed with 5% CO_2_ and 95% O_2_, 37°C, pH 7.4) and fixed in 2.5% glutaraldehyde in PBS (pH 7.4) for 1 hr at 4°C, washed and fixed in 1% osmium tetroxide, before embedding in Epon. Tissue was further subjected to postfixation with 1% osmium tetroxide and embedded in LX-112 acrylic medium. Ultra-thin cross-sections of the tracheal muscle tissue were then prepared, mounted onto coated grids, and stained with 1% uranyl acetate and lead citrate. TEM was performed with a Philips CM10, at 80 kV, on ultra thin sections (100 nm on 200 mesh grids) stained with uranyl acetate and counterstained with lead citrate.

### Intracellular Ca^2+^([Ca^2+^]_i_) measurement

Cytosolic Ca^2+^ in cultured normal (GR) and dystrophic (GRMD) cells was performed using the Ca^2+^-sensitive ratio-metric fluorescent dye Fura-2 AM as we have described previously [47], [48]. Cells grown in serum-deprived conditions (F-12+ITS) on glass coverslips or chamber slides were washed briefly with HBSS/HEPES buffer containing 0.1% BSA and then incubated with 5 µg/ml fura-2 AM (37°C, 1 h) in buffer supplemented with 0.01% pluronic acid. Cells were then washed three times and incubated in buffer for a further hour at room temperature to allow for fura-2 AM de-esterification. Real-time changes in [Ca^2+^]_i_ were recorded using an Olympus LX-70 inverted epifluorescent microscope (20x objective) coupled to a Nikon CCD camera controlled by NIS imaging software. The system was further coupled to a Sutter Instruments Lambda 10-2 filter wheel and controller with repeated 100 ms excitation at 340 and 380 nm; emission at 510 nm was recorded continually for up to 5 min after the addition of contractile agonists. Maximum change in [Ca^2+^]_i_ was calculated as the average baseline value subtracted from the peak [Ca^2+^]_i_ response to agonist. The ratio of emission at 510 nm excited by 340- and 380-nm light was converted to [Ca^2+^]_i_ values from a calibration curve generated using Ca^2+^ standards and calculated by the method of Grynkiewicz [49].

### Isolated tracheal ring preparation and measurement of Methacholine (MCh) induced isometric force

Tracheal rings were isolated using previously described method [50]. Mice were euthanized using pentobarbital overdose (90 mg/Kg bodyweight) prior to dissection. For tracheal isolation, the chest cavity contents were removed en masse and placed in Krebs-Henseleit bicarbonate solution (K-H) of the following composition (in mM): 118 NaCl, 23.5 NaCO_3_, 4.69 KCl, 1.18 KH_2_PO_4_, 1.00 MgCl_2_, 2.50 CaCl_2_, and 5.55 dextrose. The K-H was gassed with 95% O_2_-5% CO_2_ to maintain a pH between 7.3 and 7.5. Tracheal isolations were carried out in cold K-H (4°C) by pinning the apex of the heart and the voice box of trachea to a dissecting dish and removing extraneous tissue. Lungs were removed and frozen for protein analysis (see below). Each isolated trachea was cut into 4 segments; each segment containing 3 or 4 cartilage rings. Tracheal ring preparations were mounted between 2 pins- one pin firmly fixed and the other attached to an isometric force transducer in one chamber of a Danish Myo Technology (Aarhus, Denmark) organ bath system. The paries membranaceous of the tracheal ring preparation (containing the smooth muscle layer) was placed between the 2 support pins. Tissue preparations were maintained in gassed K-H at 37°C and pH 7.3–7.5 for all subsequent studies.

To establish optimal resting tension, reference length, and stable baseline tracheal rings were equilibrated for 90–120 min with intermittent (∼20 min) instillation of 63 mM KCl-substitiuted K-H (usually 3 exposures) in order to isometrically contract the tissues in a manner independent of G protein-coupled receptor activation. Reference resting tension for all preparations was established at ∼0.6 mN. The isometric force developed for each smooth muscle preparation in response to the 3^rd^ KCl-substituted K-H exposure was used as the reference force for subsequent contractions elicited through G-protein coupled receptor activation. After equilibration of the tracheal rings for 30 minutes, MCh concentration-response studies (1.0 nM to 1.0 mM) were performed. After the final administration of MCh, rings were washed with K-H.

### Measurement of Lung Mechanics

Lung mechanics was measured using a small animal ventilator as described previously [51]–[53]. After anesthetizing with Pentobarbital sodium the murine trachea was dissected using fine dissection scissors and a 20-gauge polyethylene catheter was inserted which was further connected to a flexiVent small animal ventilator (Scireq Inc. Montreal, PQ). Mice were ventilated with a tidal volume of 10-ml/kg body weight, 150 times per minute. A positive end expiratory pressure (PEEP) of 3 cmH_2_O was used for all studies. Mice were subjected to an increased dose of nebulized methacholine (MCh) challenge protocol to assess concentration response characteristics of respiratory mechanics. For MCh challenge, ∼35 µL of saline containing from 0 to 50 mg/ml MCh was delivered over 10 seconds using an in-line ultrasonic nebulizer.

To assess the effects of MCh challenge on respiratory mechanics we used a low frequency forced oscillation technique [54]. Respiratory mechanical input impedance (*Zrs*) was derived from the displacement of the ventilator’s piston and the pressure in its cylinder. Correction for gas compressibility, and resistive and accelerative losses in ventilator, tubing and catheter were performed according manufacturer instructions, using dynamic calibration data obtained from volume perturbations applied to the system in an open and closed configuration. By fitting *Zrs* to the constant phase model, flexiVent software calculated conducting airway resistance (R_aw_), peripheral tissue and airway resistance (G), tissue elastance or stiffness (H); each parameter was normalized according to body weight. Values for each parameter were calculated as the mean of all 20 perturbation cycles performed after each MCh challenge.

### Data analysis

Values reported for all data represent means ± standard error of means (SEM). For all studies, 2–3 replicate data from atleast 3–4 different plated cell cultures or animals were obtained (a total of 7–9 different experiments). The statistical significance of differences between two means was determined by an unpaired two-tailed Student’s *t*-test, or when appropriate using one way ANOVA with Bonferroni’s Multiple Comparison Test for comparison between treatments or Tukey’s multiple comparison test. Differences were considered to be statistically significant when *p*<0.05.

## Results

### Dystrophin is absent in GRMD (dystrophic) airway smooth muscle cell cultures

**Figure 1:**
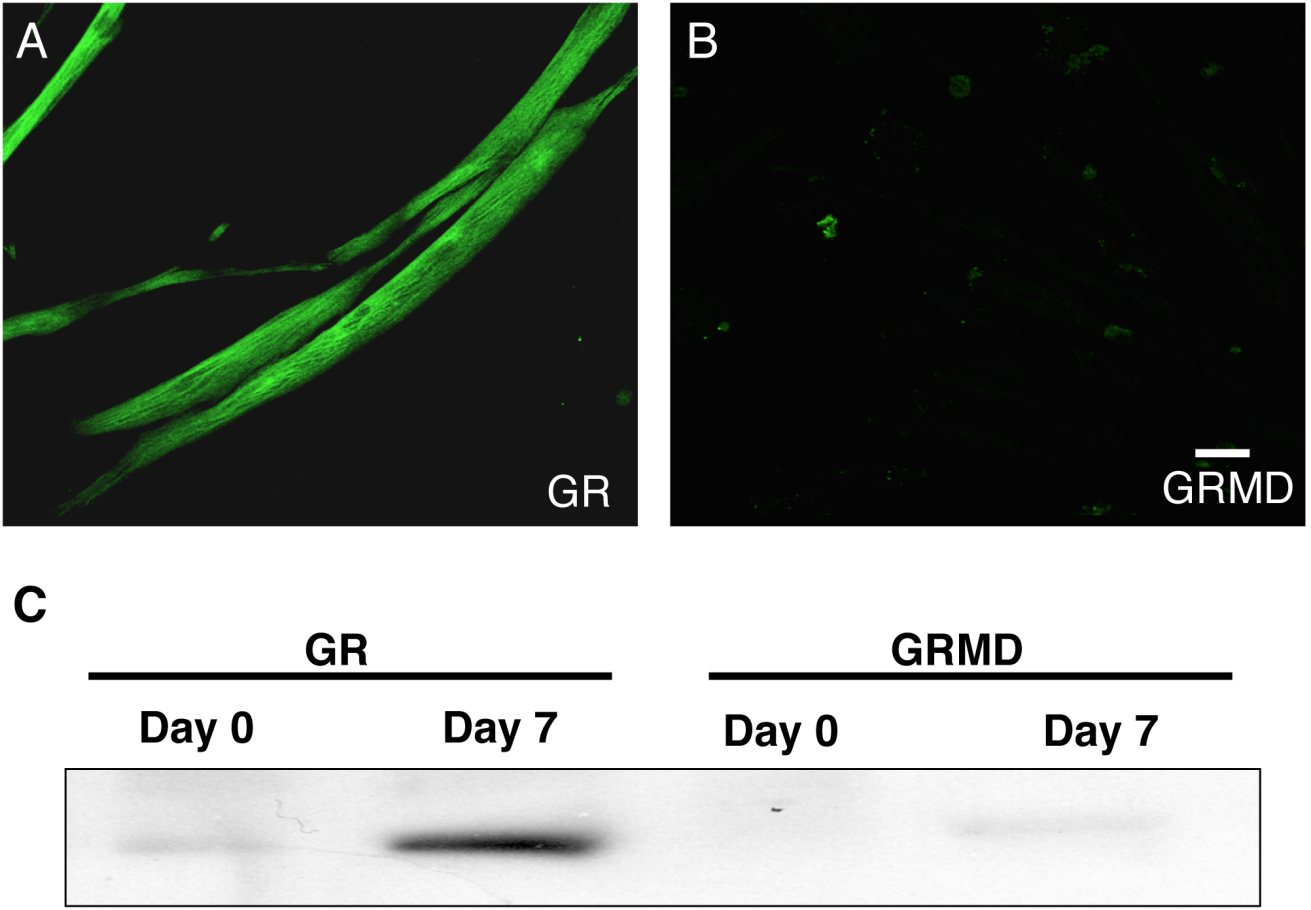
Dystrophin is absent in dystrophic ASM cells. Primary tracheal smooth muscle cells were grown to confluence from normal golden retriever (GR) (**A**) and golden retriever muscular dystrophy (GRMD) (**B**) animals and were serum starved in F12+ITS (1%) media to induce a contractile phenotype in culture. Cells were then stained for dystrophin (in green) and images were taken using a confocal laser scanning microscopy. Scale bar: 100 µm. Similarly western blotting was done for dystrophin (**C**) on primary tracheal smooth muscle cells from these animals at day 0 (proliferative phenotype) and day 7 (contractile phenotype). Results are representative of at least 6–9 *in vitro* experiments obtained from 3 different GR (normal) and GRMD (dystrophic) animals respectively.

Airway smooth muscle cells in culture have the unique capacity to acquire a long-elongated phenotype when grown in the absence of serum in ITS supplemented media [7], [45], [55]. Airway smooth muscle cells cultured from normal (GR) and dystrophic animals were subjected to serum deprivation for 7 days and were stained for dystrophin (in green) using fluorescence labeled antibodies (Fig. 1A, B). Airway smooth muscle cells derived from dystrophic animals showed negative staining for dystrophin confirming that these cells lack dsytrophin protein (Fig. 1B); while, the cells from control animal showed positive staining for dystrophin (Fig. 1A). To further confirm these results immunoblotting was performed on lysates harvested from dystrophic and normal airway smooth muscle cells at day 0 (proliferative phenotype) and day 7 (contractile phenotype) and demonstrated that airway smooth muscle cells from dystrophic animals lacked dystrophin protein (Fig. 1C).

### Dystrophin loss affects intracellular Ca^2+^ release by contractile agonist

**Figure 2:**
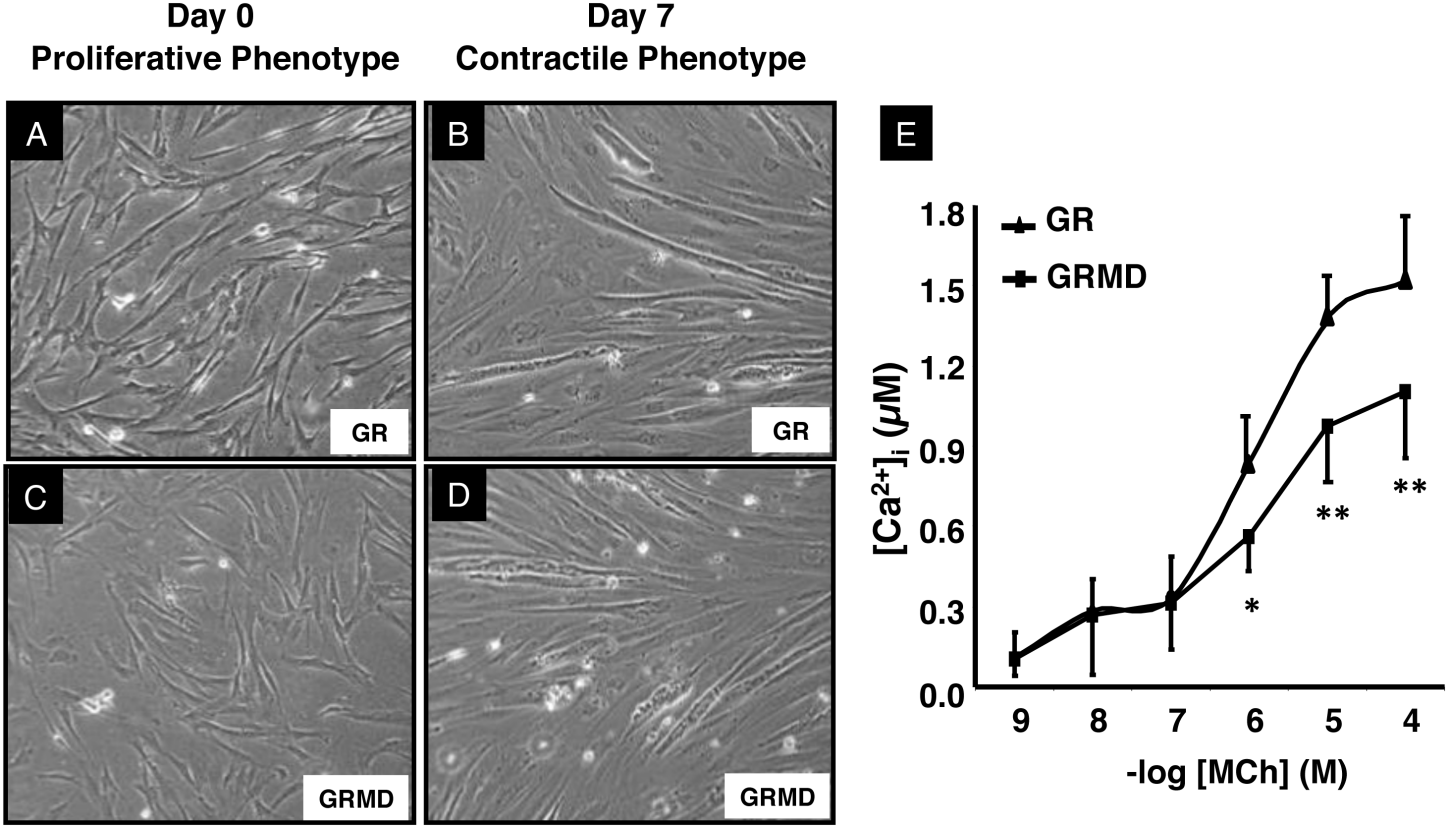
Dystrophin loss affects intracellular Ca^2+^ release by contractile agonist. Primary tracheal smooth muscle cells were grown to confluence from normal golden retriever (GR) and golden retriever muscular dystrophy (GRMD) animals and then were serum starved in F12+ITS (1%) containing media to induce a contractile phenotype in culture. Phase contrast pictures were taken at day 0 (proliferative phenotype) for GR (**A**) and GRMD (**C**) and later at day 7 (contractile phenotype) for GR (**B**) and GRMD (**D**). Images are representative of at least 6–9 different primary myocyte cultures obtained from 3 normal (GR) and dystrophic (GRMD) animals. (**E**) Representative tracings from experiments using Fura-2 loaded airway smooth muscle cells (GR and GRMD) grown to confluence then serum-deprived in insulin-supplemented media for 7 days. Cells were first stimulated with methacholine (MCh: 10^−9^ to 10^−4^ M) and changes in intracellular Ca^2+^ ([Ca^2+^]_i_) recorded. Concentration-response curves for MCh were plotted as peak [Ca^2+^]_i_. Curves are derived using individual data points that are the mean ± SEM of at least 40–45 cells in total (assayed in at least three different experiments). Statistical comparisons shown were performed by 1-way ANOVA with Tukey’s multiple comparison test. *p<0.05, **p<0.01 at a given MCh concentration.

As shown in Fig. 2, ASM cells grown in absence of serum become large and elongated after 7 days. ASM cells from dystrophic (GRMD) and normal (GR) animals were grown to confluence in DMEM supplemented with FBS (Day 0, Fig. 2A, C). Then serum was withdrawn and cells were subjected to a F12 media supplemented with ITS which promotes the contractile phenotype in culture (Day 7, Fig. 2B, D). Phase contrast pictures suggest that the characteristic shape of these flat and elongated cells lacking dystrophin was not different from the ones having functional dystrophin protein. We have shown that intracellular Ca^2+^ mobilization induced by muscarinic agonists is dependent on the organization of DGC with in caveolae [46], [47]. Using GRMD cells as a model system for disrupted DGC and loss in caveolar integrity, we measured intracellular Ca^2+^ mobilization induced by muscarinic agonist methacholine (MCh) in both normal (GR) and dystrophic (GRMD) myocytes. We found significant decrease in sensitivity to MCh in dystrophic myocytes (EC50_GR_ = 359 nM, ±25.1; EC50_GRMD_ = 744 nM, ±38.3, *p*<0.05) and reduction in maximal Ca^2+^(31.4% lower E_max_ in GRMD myocytes when compared to GR, *p*<0.05). Collectively, these results indicate that the dystrophin plays an important role in modulating receptor-mediated Ca^2+^ release in the cell.

### Effect of dystrophin on airway smooth muscle cell contractile phenotype markers

**Figure 3:**
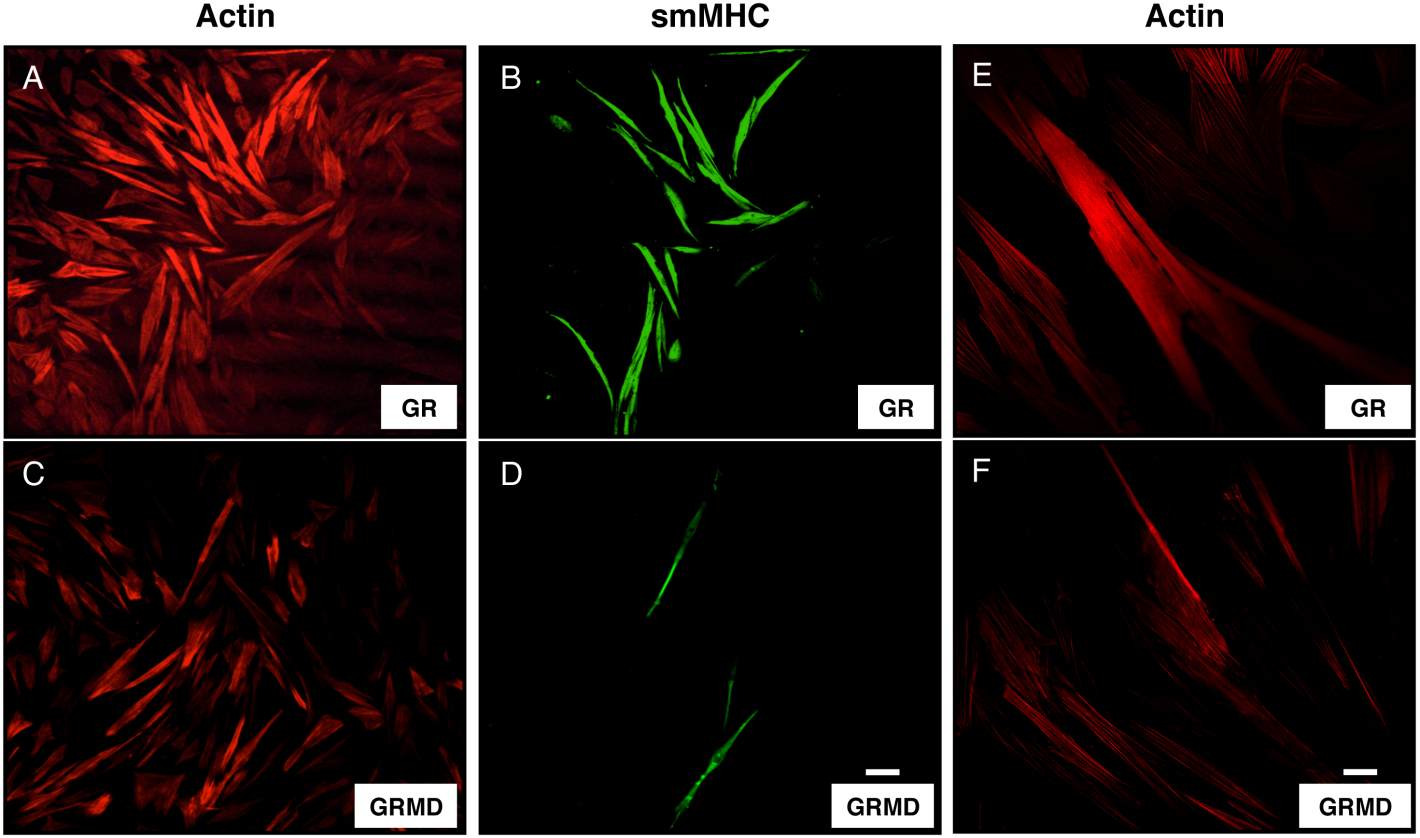
Effect of dystrophin on stress fibers and contractile marker myosin heavy chain. Primary cultured tracheal smooth muscle cells from (GR & GRMD) animals were grown to confluence on glass coverslips and subjected to 7 day culture in serum deficient conditions. Thereafter myocytes were fixed, and double labeled for (**A, C**) phalloidin, which stains f-actin, and (**B, D**) smMHC. Isotype-matched mouse IgG or rabbit antiserum was used for negative controls (not shown). Antibodies conjugated with TxR or FITC were used to label actin filaments (red) and smMHC (green) respectively. Images were obtained by confocal laser scanning microscope. Higher magnification of Fig. A & C for stress fibers are shown in panel E and F showing fluorescent phalloidin (red) marking actin filaments in GR (**E**) and GRMD (**F**). Images are representative of at least 6–9 *in vitro* experiments obtained from 3 different GR (normal) and GRMD (dystrophic) animals respectively. Scale bars, 100 µm (**A–D**) and 20 µm (**E–F**).

In canine and human ASM cells subjected to prolonged serum starvation, phenotype maturation occurs in a select subset of myocytes that become characteristically elongate, reacquire responsiveness to contractile agonists, and accumulate abundant contractile marker proteins such as smMHC, calponin, desmin and also forms a network of stress fibers [7], [45], [56], [57]. Thus, we assessed whether accumulation of these markers and stress fiber formation induced by serum deprivation was directly associated with dystrophin. Using fluorescence immunocytochemistry, after 7-day serum deprivation we double-labeled primary cultured airway smooth muscle cells (GRMD and GR) with smMHC and phalloidin (Figure 3). Consistent with previous reports [58], [59], myocytes exhibited phenotype heterogeneity, with 15–20% of ASM cells acquiring a contractile phenotype, as evidenced by increased accumulation of smMHC in these cells when compared to cells in proliferative phenotype (non-contractile). Notably, the maturation of individual ASM cells to a contractile phenotype was uniquely associated with increase in staining for actin and smMHC in normal (GR) cells having dystrophin (Figs. 3A, and 3B), whereas there was reduced staining for actin and smMHC in dystrophic (GRMD) cells (Figs. 3C and 3D) when compared to GR cells (Fig. 3A and 3B). Immunofluroscent staining data for day 0 (non-contractile) GR and GRMD cells is not shown. Moreover, qualitative assessment at higher magnification showed that stress fiber density (f-actin staining) was clearly reduced in dystrophic (GRMD) cells when compared to normal (GR) cells (Figs 3F and 3E). These data suggest that presence of dystrophin is a key determinant of dynamic actin cytoskeleton of a contractile airway smooth muscle cell. Furthermore demonstrating that at single-cell level, there is an association between the acquisition of a contractile phenotype and expression of dystrophin.

### Loss of dystrophin reduced expression of airway smooth muscle contractile phenotype markers

**Figure 4:**
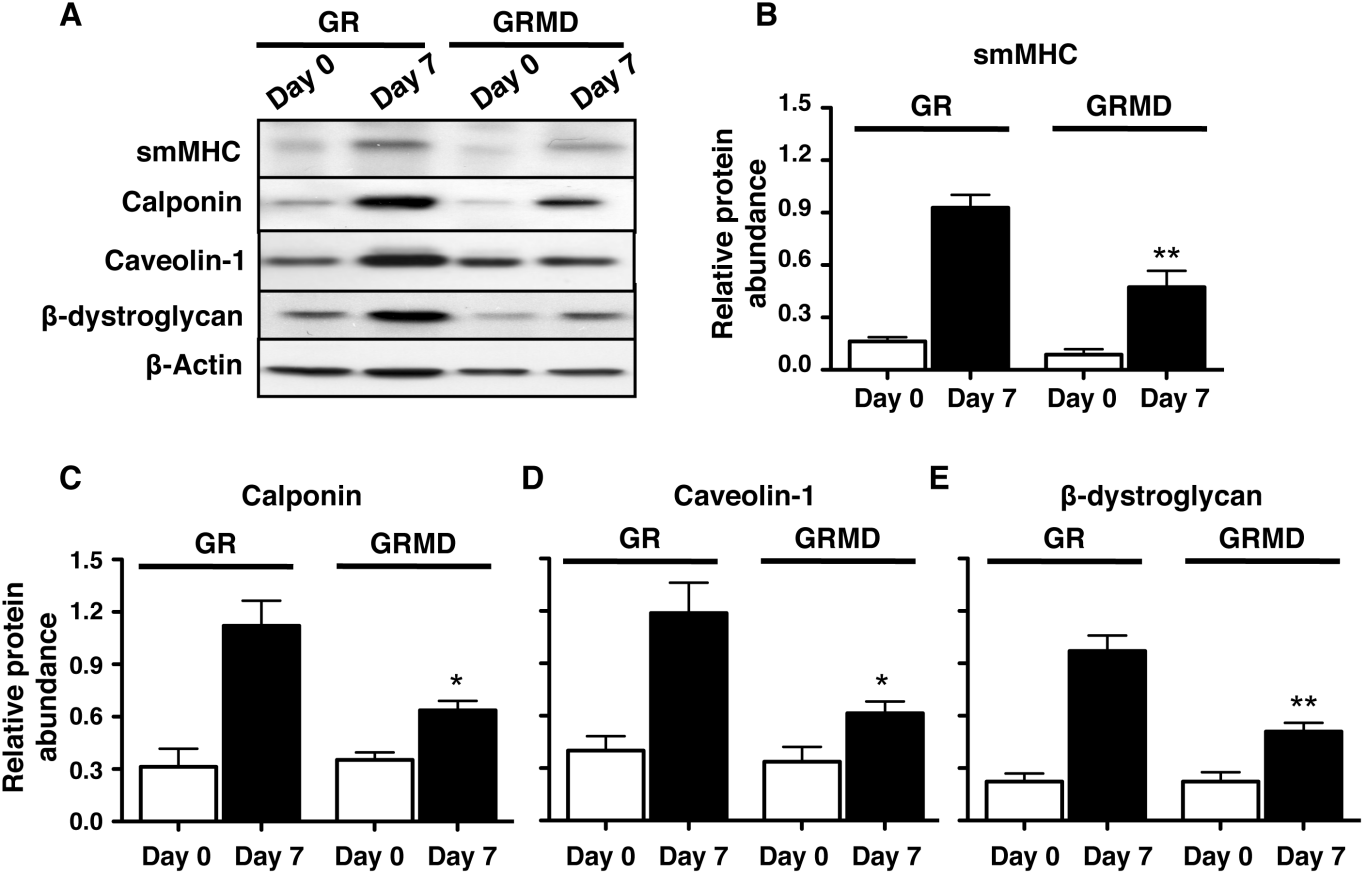
Loss of dystrophin reduces contractile protein markers. For all panels, *day 0* represents protein lysates obtained from serum-fed confluent cultures, and *day 7* represents protein lysates obtained from confluent primary tracheal smooth muscle cell cultures (obtained from GR and GRMD animals) after 7-day serum deprivation, with medium changed every 48 h. **A:** representative western blots for various contractile marker proteins under conditions described above. **B:** densitometry analysis of the effects of serum deprivation on smMHC (**B**), calponin (**C**), Caveolin-1 (**D**) and β-dystroglycan (**E**) in GR and GRMD tracheal smooth muscle cells are shown. For all histograms protein abundance was corrected for equal loading and normalized relative to β-actin abundance. Data shown represent means ± SE from 6–9 experiments using 3 different primary cells obtained from GR and GRMD animals respectively. Statistical comparisons shown were performed by 1-way ANOVA with Tukey’s multiple comparison tests. *P<0.05, **P<0.01, for GR day 7 versus GRMD day 7.

Since we showed that expression of DGC is associated with phenotype maturation of ASM cells *in vitro* [7], here we investigated whether loss of dystrophin affects the accumulation of contractile phenotype markers. ASM cells from both control (GR) and dystrophic (GRMD) animals were grown to confluence and subjected to a serum deprivation protocol for seven days. As it can be seen in the panel for western blotting (Fig. 4A) and quantification of individual proteins in Fig. 4B–E; the loss of dystrophin in GRMD cell cultures was associated with significant reduction in markers of contractile phenotype namely smMHC and calponin when compared to cells having dystrophin (GR) (Fig. 4B,C). Furthermore, there was also a significant reduction in the protein abundance of caveolin-1 and β-dystroglycan (proteins recently shown to be associated with contractile phenotype [7]). These results clearly suggest that dystrophin has a role in expression of contractile phenotype markers in ASM cells.

### Dystrophin affects PI3K signaling in airway smooth muscle cells

**Figure 5:**
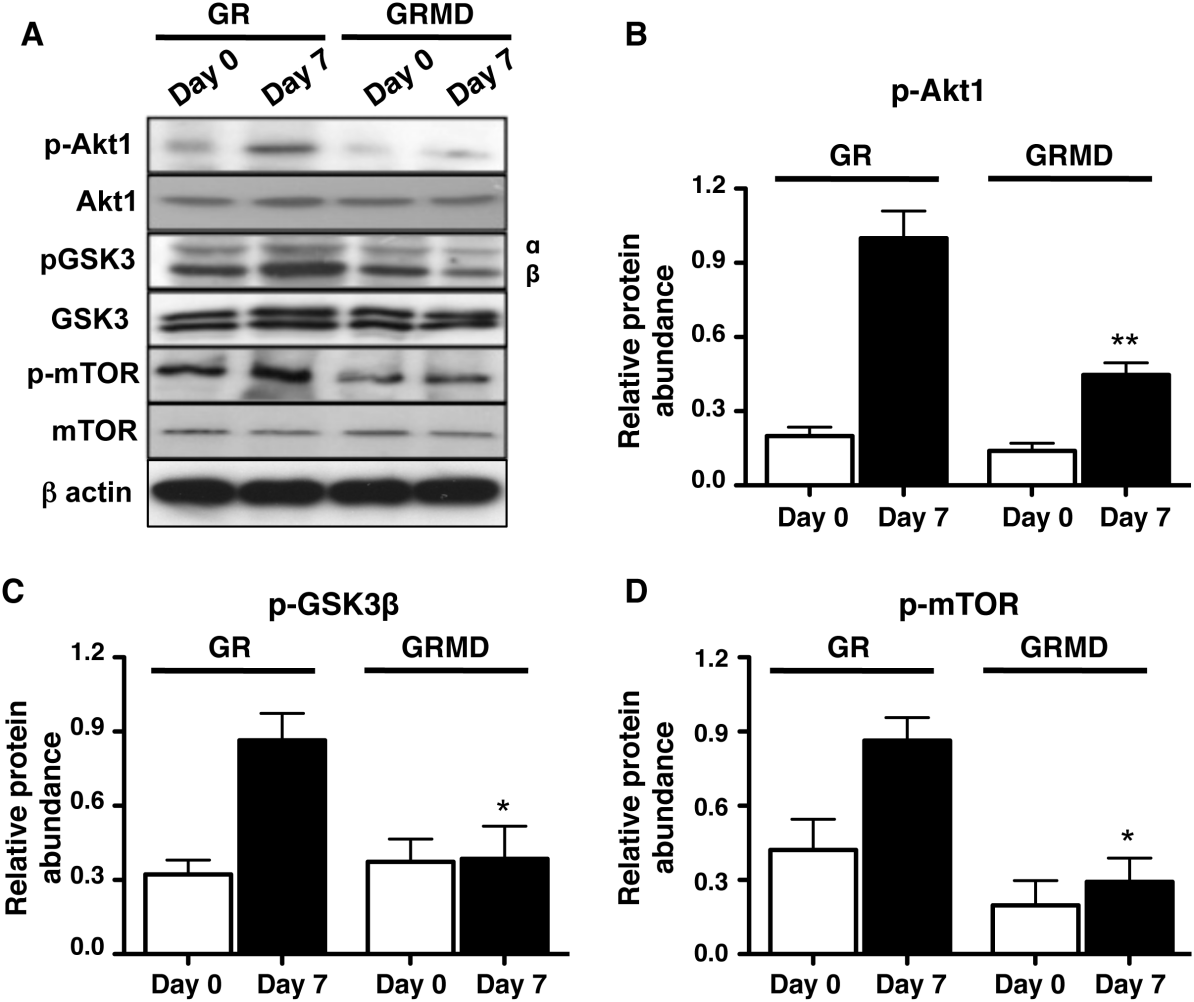
Loss of dystrophin reduces induction of PI3K-signaling. For all panels, *day 0* represents protein lysates obtained from serum-fed confluent cultures, and *day 7* represents protein lysates obtained from confluent primary tracheal smooth muscle cell cultures (obtained from GR and GRMD animals) after 7-day serum deprivation, with medium changed every 48 h. **A:** representative western blots for PI3K-GSK3-mTOR signaling pathway proteins. **B:** densitometry analysis of the effects of serum deprivation on p-Akt1 (**B**), p-GSK3β (**C**) and p-mTOR (**D**) in GR and GRMD tracheal smooth muscle cells are shown. For all histograms β-actin was used as a loading control and phospho-proteins were normalized relative to their respective total protein. Data shown represent means ± SE from 6–9 experiments using 3 different primary tracheal smooth muscle cells obtained from healthy (GR) and dystrophic (GRMD) animals. Statistical comparisons shown were performed by 1-way ANOVA with Tukey’s multiple comparison tests. *p<0.05, **p<0.01, for GR day 7 versus GRMD day 7.

Previous studies have demonstrated that signaling through the phosphatidylinositide-3-kinase (PI3K) pathway, including Akt1, p70S6 kinase, and mTOR, is required for ASM maturation, hypertrophy, and concomitant accumulation of contractile protein markers [7], [56], [60]. Here we investigated whether dystrophin affects PI3K signaling in airway smooth muscle cells. Similarly to the previous figure, airway smooth muscle cells were subjected to serum deprivation protocol for 7 days and lysates were collected at day 0 and day 7. As seen in the panel for westerns (Fig. 5A), loss of dystrophin was associated with the reduction of activation of key signaling proteins namely p-Akt-1, p-GSK3β and p-mTOR. Densitometric analysis showed that the reduction in the phosphorylation of these signaling proteins in cells lacking dystrophin (GRMD) was significant when compared to the ones having normal dystrophin expression (GR) (Fig. 5B–D). These results clearly demonstrate that dystrophin is associated with the expression of contractile phenotype and also regulate the key signaling machinery required for this process to occur *in vitro*.

### Dystrophin affects caveolar integrity and airway smooth muscle contraction

**Figure 6:**
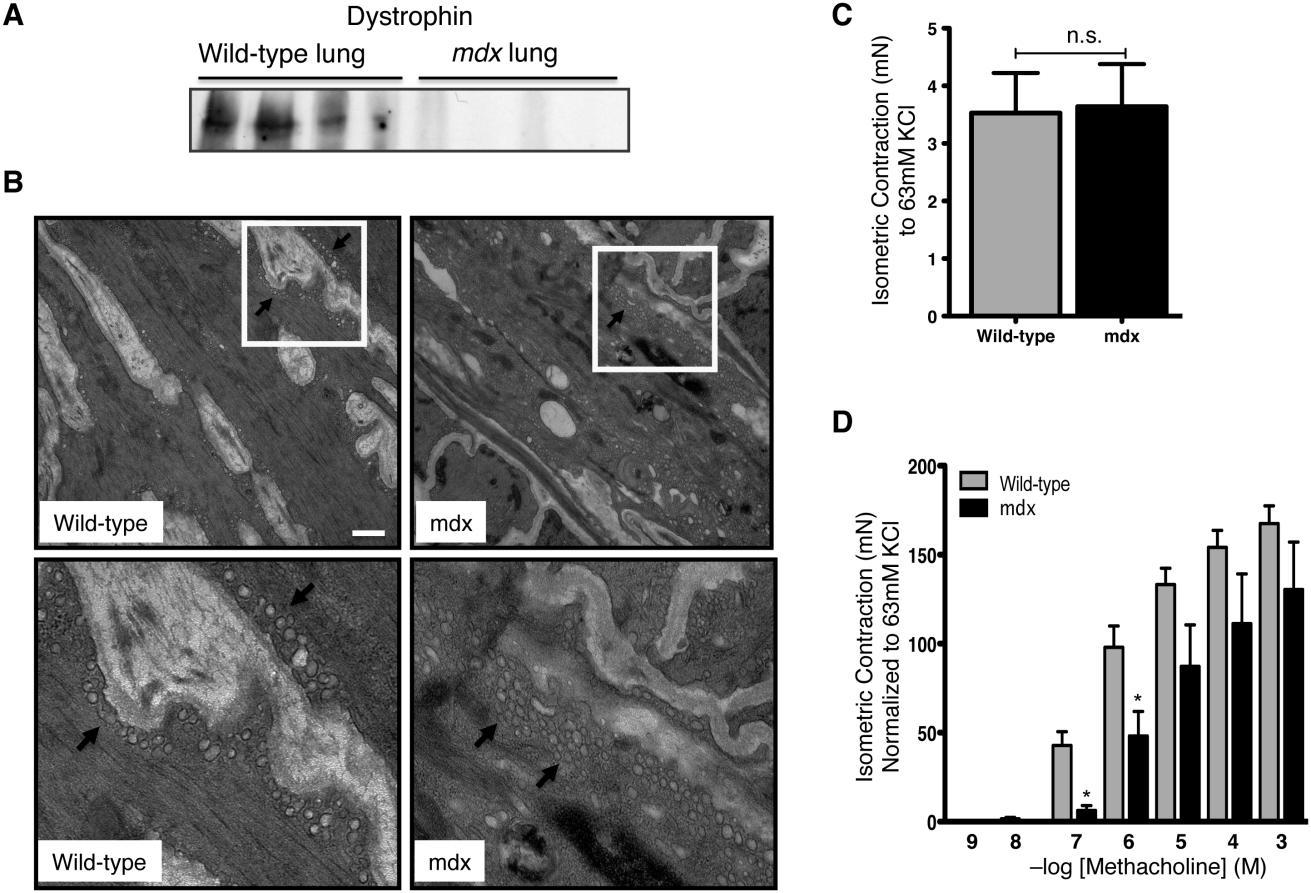
Loss of dystrophin reduces tracheal contractility. **A:** representative western blots typical of those obtained for 4 different *mdx* mice lungs (aged 8–12 weeks) showing dystrophin protein is absent in *mdx* mice. **B:** Tracheal tissues from wild type or *mdx* mice were isolated and then fixed for transmission electron microscopy. Arrows in upper and lower left panels (wild-type) indicate typical caveolae; whereas in upper and lower right panels, arrows indicate internalized caveolae like double-membrane structures that appear more in *mdx* mice as seen in the lower right panel. Scale bar: 100 nm. Tracheal rings from at least 4–6 mice were used to obtain the above results. Comparing the 2 groups, unpaired t-test indicated a *p-value of* 0.9136; *n.s.* **C:** tracheal rings from *mdx* and wild-type mice were isolated and equilibrated for 90–120 min with intermittent (∼20 min) instillation of 63 mM KCl-substituted K-H to obtain a resting tension at ∼0.6 mN. Concentration response studies with methacholine demonstrated significantly reduced responsiveness of *mdx* preparations at lower concentrations compared to wild-type (**D**). Statistical comparisons shown were performed by 1-way ANOVA with Tukey’s multiple comparison tests. *P<0.05, for *mdx* versus wild-type.

To further support our conclusion from the biochemical data (Fig. 4A) and test whether dystrophin loss affects caveolar organization we performed transmission electron microscopy (TEM) in tracheal tissue obtained from *mdx* (dystrophin KO) and wild-type mice. The qualitative assessment of the ultra-structural details demonstrate that caveolar invaginations on the tracheal smooth muscle membrane were markedly reduced in the *mdx* mice and there is a greater tendency for the these structures to internalize in absence of dystrophin when compared with wild-type (Fig. 6B). To further assess the role of dystrophin in determining the functional responses such as airway smooth muscle contraction *ex vivo*, we employed mice having a spontaneous mutation in dystrophin gene (*mdx* mice) [8], thus lacking functional dystrophin protein. As shown in Fig. 6A the lung homogenates from 4 *mdx* mice showed no dystrophin when compared to the genetic controls BL10 (wild-type) mice. We then studied the isometric contraction of tracheal rings obtained from these mice. There was no change in the development of basal isometric force induced by KCl in *mdx* and wild-type mice (Fig. 6C). The cumulative dose-response-curve to a contractile agonist methacholine (MCh) in *mdx* and wild-type trachea showed that there was a significant reduction of isometric force at submaximal and sub-EC_50_ concentrations of MCh (Fig. 6D). The sensitivity to MCh was reduced significantly in *mdx* mice trachea as evident by increase in the EC_50_ values (EC_50wild-type_ = 0.519 µM ±0.098 as compared to EC_50*mdx*_ = 2.48 µM ±0.16) of *mdx* mice (*p*<0.05).

### Role of dystrophin on lung physiology

**Figure 7:**
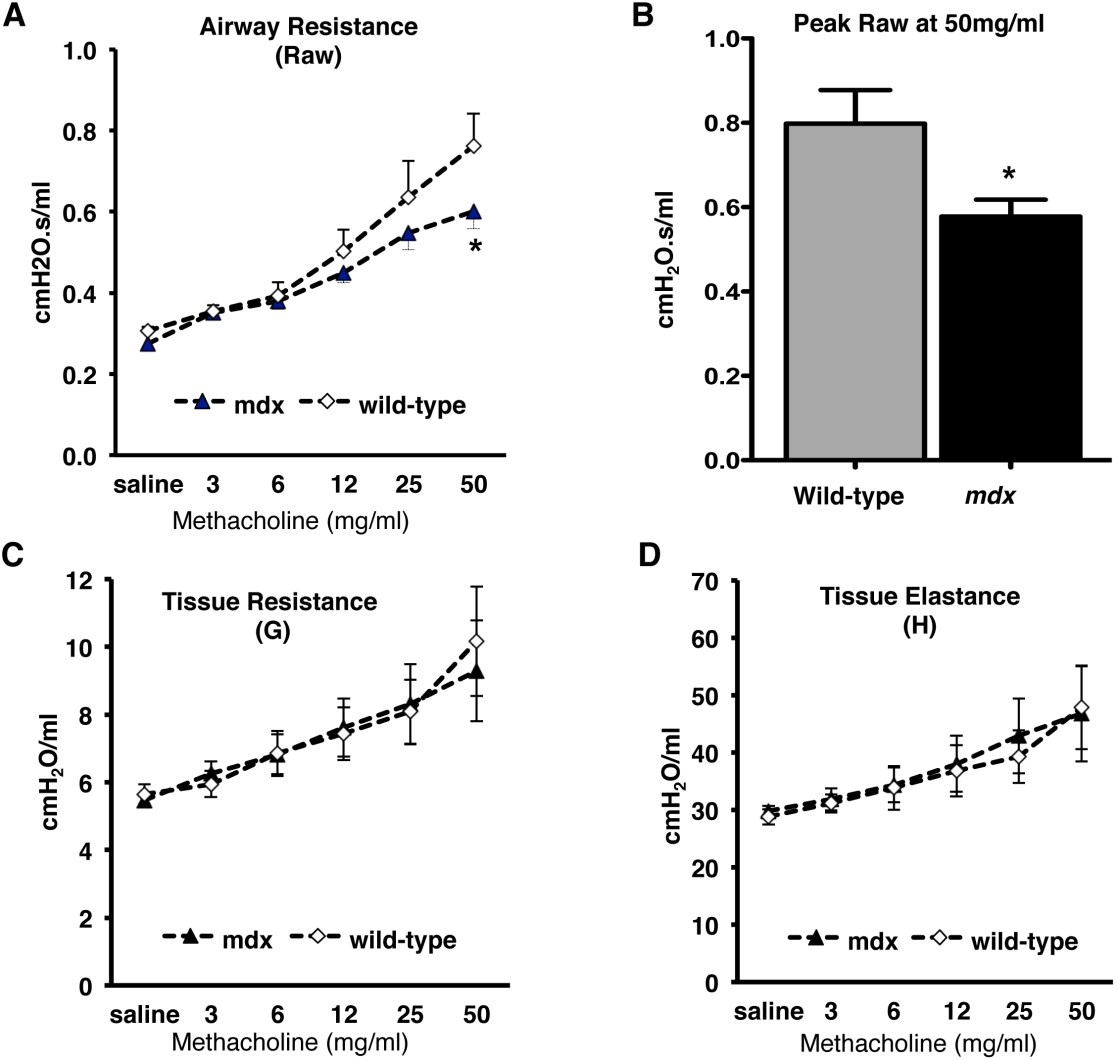
Effect of dystrophin on lung function. Wild-type or *mdx* mice tracheas were dissected and connected to a flexiVent small animal ventilator (Scireq Inc. Montreal, PQ). Mice were ventilated with a tidal volume of 10 ml/kg body weight, 150/minute. Mice were then subjected to an increased dose of nebulized methacholine (MCh) challenge protocol to assess characteristics of respiratory mechanics. For MCh challenge, ∼30 µL of saline containing from 0–50 mg/ml MCh was delivered over 10 seconds using an in-line ultrasonic nebulizer. By fitting respiratory mechanical input impedance (*Zrs*) to the constant phase model, flexiVent software calculated conducting airway resistance (Raw) (**A**), peak Raw at 50 mg/ml of MCh (**B**), peripheral tissue and airway resistance (G) (**C**), tissue elastance or stiffness (H) (**D**); each parameter was normalized according to body weight. Values for each parameter were calculated as the mean of all 20 perturbation cycles performed after each MCh challenge. Statistical comparisons shown were performed by 1-way ANOVA with Tukey’s multiple comparison tests; *P<0.05, was considered significant for *mdx* versus wild-type (unpaired t-test performed in Fig. B). Data shown is the ±mean of 9–10 *mdx* and wild-type mice.

Finally to determine the physiological role of dystrophin we used *mdx* and wild-type mice and performed lung function using a small animal ventilator (flexiVent). Airway resistance (Raw), tissue resistance (G) and tissue elastance (H) was determined using increasing doses of nebulized MCh. The peak airway resistance (at 50 mg/ml MCh) was reduced significantly (∼25%; *p*<0.05) in *mdx* mice when compared to the wild-type (Fig. 7B) while there was no significant reduction at lower concentrations of MCh (Fig. 7A). In contrast, no differences in tissue resistance (Fig. 7C) and elastance (Fig. 7D) between the two mice strains were observed. These results indicate that dystrophin expression is an important determinant of maximal airway constriction, and are suggestive that dystrophin may broadly modulate lung physiology and airway responsiveness.

## Discussion

We have shown that dystrophin glycoprotein complex (DGC) subunits are abundantly expressed in contractile airway smooth muscle cells and tissue, and their expression is associated with phenotype switching *in vitro* [7]. Moreover, β-dystroglycan - a central subunit of DGC - interacts directly with caveolin-1 in airway smooth muscle cells, and this interaction is important for mobilization of intracellular calcium induced by contractile agonists [46]. Caveolae microdomains in airway smooth muscle cells harbor key signaling proteins required for mobilization of intracellular calcium and caveolin-1 plays an important role in modulating airway smooth muscle phenotype and function [47], [61]–[65]. In other smooth muscle types, dystrophin colocalizes with caveolin-1 and occupies complementary distribution with adheren junctions [5]. Thus, we investigated its role in airway smooth muscle phenotype and function. Our data indicate that dystrophin expression is associated with a contractile phenotype in airway smooth muscle cells, and its absence is associated with suppression of phenotype maturation. Notably, loss of dystrophin reduced the activation of the PI3K-Akt-mTOR signaling pathway, which is required for accumulation of contractile protein markers. Our current study demonstrates that dystrophin has an important role in airway smooth muscle contraction and it provides a first insight into its role in lung physiology.

The plasma membrane of contractile smooth muscle cells is highly ordered, consisting of repeating longitudinal rib-like arrays of caveolae microdomains and adherens junctions [6], [66]–[69]. North and colleagues [5] provided initial evidence that caveolae microdomains are marked by the presence of both caveolin-1 and dystrophin. This has contributed to the understanding of the organization of cytoskeleton in smooth muscle but there has been no investigation assessing the exact role of dystrophin in a contractile airway smooth muscle cell, both in terms of phenotype and function. Dystrophin along with other DGC subunits are markers of a contractile phenotype in airway smooth muscle [7]. Our new data using dystrophic animals show that dystrophin loss is associated with the concomitant reduction of contractile protein markers upon phenotype switching. This report is the first to describe a role for dystrophin in phenotype maturation and functional responses of airway smooth muscle cells and tissue. We recently described that the actin cytoskeleton plays an important modulator role in calcium homeostasis, and suggested that this may in part be facilitated by dystrophin and β-dystroglycan that provide a transmembrane link between ECM and underlying actin cytoskeleton [46]. Our current studies with dystrophin deficient airway smooth muscle cells demonstrate that dystrophin is crucial for the formation of stress fibers in these cells as lack of dystrophin is associated with a marked reduction in the stress fibers formation. Our *in vitro* data reveals that the permanent loss of dystrophin (as in GRMD myocytes) resulted in reduction in key caveolar protein marker (caveolin-1) along with other contractile protein markers (calponin) and a central DGC subunit (β-dystroglycan). We have shown [46] that caveolin-1 and DGC binding (through β-dystroglycan) are integral to the organization and structural integrity of membrane caveolae and provides a strong link between ECM protein and the actin cytoskeleton, the dynamics of actin cytoskeleton in turn regulate variety of cell responses. Our data test this paradigm as in dystrophin deficient ASM cells (GRMD), we found reduced responsiveness to receptor-mediated Ca^2+^ release clearly pointing that link between dystrophin and actin provides structural integrity to caveolae which drives receptor-mediated responses of the cell.

PI3K-signaling is critical for contractile phenotype maturation, and for myocyte elongation and hypertrophy of airway smooth muscle cells [56], [60]. This pathway is also important in skeletal muscle, because myotube hypertrophy and the accumulation of contractile proteins require PI3K-Akt1-mTOR and PI3K-Akt1-GSK3β signal transduction pathways [70], [71]. We have shown that accumulation of dystrophin along with other DGC subunits is regulated by mechanisms such as laminin-integrin binding and induction of PI3K-signaling in ASM cells [7]. Here we investigated whether loss of dystrophin affects the PI3K signaling during contractile phenotype acquisition. Our results show that loss of dystrophin results in suppression of phosphorylation of signaling effectors downstream PI3K eg. Akt1, GSK3β and mTOR one of the contributing mechanisms are by altering the structural integrity of caveolar structures as seen in *mdx* tracheal muscle (increased internalization) by transmission electron microscopy. These results are suggestive that dystrophin does not as a direct signaling effector but indirectly modulate PI3K-signaling (through caveolae) which is an essential signaling pathway for phenotype maturation of airway smooth muscle cells. That there appears to be no change in contractile apparatus organization in *mdx* mice may be due to the prolonged nature of dystrophin loss in the animals-meaning that compensatory mechanisms that are not tapped *in vitro* (when cells are cultured acutely) can be invoked. In case of *mdx* mice (a milder phenotype of DMD), the ASM pathology is not severe, likely due to adaptive changes such as increased expression of utrophin which is 7% shorter than dystrophin but with similar structure and function [72]–[74]. Our current study does have the limitation that we did not investigate this in detail as no suitable antibodies could be obtained, however our prior work [7] did reveal utrophin mRNA is present in cultured contractile human ASM cells.

The DGC complex plays an important role in stabilizing skeletal muscle fibers, providing support to the muscle during repeated contraction and relaxation [75]. Studies are lacking that directly assess the role of the DGC or dystrophin per se in mechanical load bearing in smooth muscle cells, though a recent report from Dye and colleagues [76] reveals that carotid arteries from mdx and δ-sarcoglycan knock out mice exhibit decreased pressure-induced distensibitity. The absence of dystrophin in portal veins from *mdx* mice leads to reduce stretch-induced myogenic contractile responses [77]. Notably, ectopic smooth muscle-specific expression of dystrophin can improve aberrant vasoregulation in mdx mice [78]. Morel et al [79] reported that decreased mechanical activity of duodenal smooth muscle in mdx mice is due to reduced type 2-ryanodine receptor expression that compromises sarcoplasmic reticulum calcium release. Cohn et al [80] showed that cardiac myopathy associated with coronary artery vasospasm in sarcoglycan knock out mouse models could be prevented by verapamil, a general Ca^2+^ channel blocker with vasodilatory properties.

These above observations prompted us to investigate the role of dystrophin in contraction of tracheal smooth muscle isolated from *mdx* mice. Our results indicate that dystrophin contributes to maintaining tracheal smooth muscle sensitivity to contractile agonists, as loss of dystrophin in *mdx* mice was associated with reduced sensitivity of mouse tracheal rings at submaximal concentrations of MCh without affecting the maximal contractile response. Interestingly receptor-independent contraction to KCl was unaffected in *mdx* mice tracheal rings which is somewhat in-line with our previous work using caveolin-1 KO mice [81] where we found that epithelium-derived mediators can modulate tracheal smooth muscle responsiveness to contractile agonists at least in some instances (when COX-2 is inhibited). Another likely explanation for this differential effect is the presence of muscarinic receptor reserve that has been documented in ASM [82], [83], rendering responses to higher MCh concentrations (>1 µM) refractory to any reduction in coupling to G proteins and/or other downstream signaling effectors. Our findings in this MS suggest that the receptor independent force generating capacity of *mdx* tracheal rings remains unchanged but there are differences in the receptor-mediated force generation capacity of tracheal rings (at sub EC_50_ and submaximal concentrations) to MCh. Our new findings suggests that alternate mechanisms to alter contractility may be enhanced with long term dystrophin depletion as in *mdx* mice. The ultra-structural details obtained from *mdx* mice trachea revealed that dystrophin loss resulted in the internalization of caveolar structures. This qualitative assessment clearly demonstrates that caveolae invaginations on the tracheal smooth muscle membrane are markedly reduced in tissue from *mdx* mice. Moreover, there is a redistribution of caveolae-like structures, as they appear to be internalized in absence of dystrophin. We agree that there is a limitation of this analysis, as we did not perform immunogold staining for caveolin-1. However, our previous work has shown strong correlation between the internalization of membrane caveolae with the reduced expression of contractile protein markers and key-signaling molecules (PLCβ1 and Gαq) required for receptor-mediated Ca^2+^ release [46], [47]. We have also showed that trafficking of caveolin-1 protein along with DGC subunits from caveolae-rich fractions to non-caveolae fraction is affected when tethering of actin to dystrophin was perturbed [46].

A great deal of what is known about dystrophin structure-function has come from studies of a variety of dystrophin-deficient animals [17], [23], but by far the most prolific model has been the *mdx* mouse, first described in 1984 by Bulfield et al [16]. The skeletal muscles of *mdx* mice show a marked susceptibility to lengthening contraction-mediated force decrements [84]–[87]. Contractile dysfunctions are also evident in cardiac muscles of the *mdx* mouse [88], [89]. These observations suggest that dystrophin is important in excitation-contraction coupling of skeletal and cardiac muscles and lead us to investigate its role in lung physiology. Our results with *mdx* mice are suggestive of the fact that dystrophin might affect airway and lung function. We observed a statistically significant reduction in airway resistance in *mdx* mice at the highest concentration of aerosolized MCh, while at lower MCh concentrations these values failed to achieve statistical significance. Notably for our work we used 8-week old young adult mice. The nature of our findings and knowledge that the dystrophic muscle phenotype is progressive in nature [20]–[22] suggest a need for future studies in which lung function is followed as mice age past 8 weeks.

In summary, our study investigated the role of dystrophin in phenotype maturation of ASM cells and the association of dystrophin deficiency with activity of signaling proteins required for development of a contractile phenotype *in vitro*. *Ex vivo* tracheal smooth muscle contraction data using *mdx* mice clearly show that dystrophin is required for generating contractile force in response to muscarinic agonist. These findings did not translate completely to the intact animal where we found no significant decline of lung function in the *mdx* mice at lower concentrations of MCh, although we did observe a significant reduction in the airway resistance at the highest concentrations of MCh. Collectively, our data suggests that further studies are warranted to assess the impact of aging on airway physiology in dystrophin deficient models as the results presented in the current study were done on young *mdx* mice.
